# Testing the Potential for Predictive Modeling and Mapping and Extending Its Use as a Tool for Evaluating Management Scenarios and Economic Valuation in the Baltic Sea (PREHAB)

**DOI:** 10.1007/s13280-013-0479-2

**Published:** 2014-01-12

**Authors:** Mats Lindegarth, Ulf Bergström, Johanna Mattila, Sergej Olenin, Markku Ollikainen, Anna-Leena Downie, Göran Sundblad, Martynas Bučas, Martin Gullström, Martin Snickars, Mikael von Numers, J. Robin Svensson, Anna-Kaisa Kosenius

**Affiliations:** 1 Department of Biological and Environmental Sciences, Tjärnö, 45296 Strömstad, Sweden; 2 Department of Aquatic Resources, Swedish University of Agricultural Sciences, Skolgatan 6, 74242 Öregrund, Sweden; 3 Environmental and Marine Biology & Husö Biological Station, Åbo Akademi University, Tykistökatu 6, 20520 Turku, Finland; 4 Coastal Research and Planning Institute, Klaipeda University, 92294 Klaipeda, Lithuania; 5 Department of Economics and Management, University of Helsinki, P.O. Box 27, 00014 Helsinki, Finland; 6 Centre for Environment, Fisheries and Aquaculture Science, Pakefield Road, Lowestoft, Suffolk, NR33 0HT UK; 7 AquaBiota Water Research, 115 50 Stockholm, Sweden; 8 Department of Ecology, Environment and Plant Sciences, Stockholm University, Stockholm, Sweden; 9 Department of Biosciences, Environmental and Marine Biology, Åbo Akademi University, Tykistökatu 6, 20520 Turku, Finland; 10 School of Biological Sciences, Monash University, Clayton, VIC 3800 Australia; 11 Pellervo Economic Research PTT, Eerikinkatu 28 A, 00180 Helsinki, Finland

**Keywords:** Predictive mapping, Management scenarios, Benthic habitats, Economic valuation, Baltic Sea

## Abstract

We evaluated performance of species distribution models for predictive mapping, and how models can be used to integrate human pressures into ecological and economic assessments. A selection of 77 biological variables (species, groups of species, and measures of biodiversity) across the Baltic Sea were modeled. Differences among methods, areas, predictor, and response variables were evaluated. Several methods successfully predicted abundance and occurrence of vegetation, invertebrates, fish, and functional aspects of biodiversity. Depth and substrate were among the most important predictors. Models incorporating water clarity were used to predict increasing cover of the brown alga bladderwrack *Fucus vesiculosus* and increasing reproduction area of perch *Perca fluviatilis*, but decreasing reproduction areas for pikeperch *Sander lucioperca* following successful implementation of the Baltic Sea Action Plan. Despite variability in estimated non-market benefits among countries, such changes were highly valued by citizens in the three Baltic countries investigated. We conclude that predictive models are powerful and useful tools for science-based management of the Baltic Sea.

## Background

Marine and coastal ecosystems contribute substantially to the global pool of biodiversity and production of goods and services (Costanza et al. 1997). These ecosystems are increasingly exploited and consequently human pressures are now heavily affecting most coastal seas on a global scale (Halpern et al. 2008; HELCOM 2010; OSPAR 2010). The semi-enclosed and species poor Baltic Sea, with its densely populated catchment area is no exception. Recent assessments have shown that biodiversity and ecosystem functioning, including essential ecosystem services, in the Baltic Sea are currently severely affected (HELCOM 2010; BalticSTERN 2013).

Current impacts and predicted future increases of human pressures have led to a number of agreements and legislative efforts affecting the management of European coastal seas, for example, the Baltic Sea Action Plan (BSAP), Marine Strategy Framework Directive (MSFD), and a recently proposed directive for maritime spatial planning and integrated coastal management (MSP). All of these initiatives emphasize actions, adaptivity, and the need for integrating ecological and socio-economic systems. It is also evident that these frameworks pose several new methodological challenges, for example, how to make status assessments, how to detect and evaluate human impacts, and how to achieve integrated planning. The project PREHAB (Spatial PREdiction of benthic HABitats in the Baltic Sea: incorporating anthropogenic pressures and economic evaluation) was designed to address one particular challenge relevant in many management contexts: the need for comprehensive and spatially explicit information about structural and functional aspects of biodiversity in the marine environment.

Maps and other information of areal extent are essential for assessing the status according to the MSFD and for implementing future MSP directives. A major difficulty is that data on vegetation, invertebrates, and fish in the marine environment are very scattered and sparse. This is because they are usually collected using dives, corers, videos, or nets. To develop comprehensive distribution maps, methods that predict the state in unsampled sites by integrating existing data are needed (Li and Heap 2008). Such predictive methods, based on statistical species–environment relationships and often called habitat- or species distribution models (SDMs), are increasingly used in both terrestrial and marine contexts (for some recent examples, see Reiss et al. 2011; Sundblad et al. 2011; Gonzalez-Mirelis and Lindegarth 2012; Nyström Sandman et al. 2012; Downie et al. 2013). A wide array of methods, including generalized linear and additive models, tree-based methods, and machine learning techniques or combinations thereof, are now available for SDMs and the number of statistical approaches is growing (Guisan and Zimmerman 2000; Segurado and Araújo 2004; Elith et al. 2006; Araújo and New 2007). These methods are typically very flexible in terms of quantifying nonlinear relationships and interactions among predictors, but also with respect to predicting quantitative or categorical response variables, that is, dealing with regression and classification problems. While successful predictive modeling requires ecological understanding of the species of interest and careful selection of pertinent and powerful predictor variables (Guisan and Thuiller 2005; Elith and Leathwick 2009), these methods are essentially correlative. Furthermore, the great flexibility of these methods means that many types of available data of varying quality are often included when they are applied in practice.

One major challenge within PREHAB was to use typical datasets from different parts around the Baltic Sea to evaluate systematically which aspects of biodiversity can be predicted (e.g., abundance or occurrence of different organism groups), which predictors are generally powerful and which methods generally perform well. This would be a foundation for evidence-based recommendations on biodiversity mapping to authorities in the Baltic Sea region. Furthermore, the aim was to explore how the use of SDMs could be extended into evaluation of ecological and economic effects of management actions. The rationale and results of these efforts are briefly summarized below.

## Rationale and Overall Approach

The scientific activities of PREHAB were structured into three main parts: (1) evaluating the potential for predictive modeling in the Baltic Sea, (2) modeling responses to management scenarios, and (3) monetary valuation of management scenarios.

First, the scientific core of PREHAB was a systematic assessment of the general applicability of predictive, empirical SDMs in the Baltic Sea (Bučas et al. 2013). Using datasets from five case-study areas and a range of modeling techniques, we evaluated predictive performance of models across the Baltic Sea. We assessed models of quantitative responses (i.e., abundance or percent cover) using generalized additive models (GAM; Hastie and Tibshirani 1990), multivariate adaptive regression splines (MARS; Friedman 1991), and random forests (RF; Breiman 2001). For occurrence (i.e., models of presence vs. absence), we additionally used the maximum entropy method (MAXENT; Phillips et al. 2006).

Second, we developed and illustrated approaches where SDMs can be used to link human pressures or management actions to landscape-level ecosystem functions (Bergström et al. 2013) and ultimately to economic values. This was done by incorporating an important indicator of eutrophication, i.e., Secchi depth, as a predictor in the distribution models of vegetation and fish recruitment (another study explored the links between shoreline exploitation and fish recruitment, as well as the link between recruitment habitat and stock sizes; Sundblad et al. 2013). Using observed, quantitative relationships between Secchi depth and the distribution of coastal vegetation and fish, we used ensemble methods to assess potential habitat distribution changes due to a range of different management scenarios relating to the BSAP.

Finally, the predicted changes in habitat distributions, as expected after fulfillment of the BSAP, were used as a fundament to design an economic valuation study (Kosenius and Ollikainen 2011). The monetary valuation was based on the willingness-to-pay method (e.g., Champ et al. 2003) using questionnaires distributed to panels in Finland, Lithuania, and Sweden.

## Overview of Results

### Evaluating the Potential for Predictive Modeling in the Baltic Sea

More than 5000 biological samples and a total of 77 response variables across different levels of taxonomy were used for evaluating the potential for distribution modeling in the Baltic Sea (Bučas et al. 2013). The response variables consisted of a wide variety of taxa including species, higher taxonomic level groups (e.g., Hydroidea, Phanerogams), groups assigned according to their functional form (e.g., filamentous algae, sessile filter feeders), and species richness. The responses were grouped into macrophytes, macrozoobenthos, fish, and species richness. We examined a variety of environmental variables to assess their usefulness in predictive modeling of abundance and distribution patterns of benthic species and habitats. Based on known species–environment relationships in the Baltic Sea (reviewed by Snickars et al. 2014), a number of relevant predictor variables were selected and classified into five main categories: geographical location (e.g., longitude and latitude), bottom topography (e.g., depth, curvature, and aspect), wave exposure (at surface and depth-attenuated), bottom substrate (e.g., cover of rocky, non-mobile and soft, mobile), and hydrography (e.g., salinity, temperature, pH, and Secchi depth).

This resulted in approximately 200 quantitative and 300 occurrence models of vegetation, benthic invertebrates, fish, and indices of biodiversity from a wide range of environmental conditions representing different parts of the Baltic Sea. These models were used to assess (1) overall predictability of benthic species and habitats, (2) differences in performance among modeling approaches (GAM, RF, MARS, and MAXENT), (3) differences in predictability among types of organisms, and (4) differences in predictive power among different types of predictors. Each model was assessed against a test dataset (30 % of all data), which was left out at the training stage. Area under the receiver operating characteristic curve (AUC) values were used to validate models of presence versus absence, whilst the coefficient of determination (*R* ^2^), and root mean square error (RMSE) were used for abundance models. RMSE values were normalized to the range of each response (NRMSE). In addition to these analyses, we also developed a strategy for assessing (5) the importance of spatial resolution in one of the case-study areas.

#### Overall Predictability

**Fig. 1:**
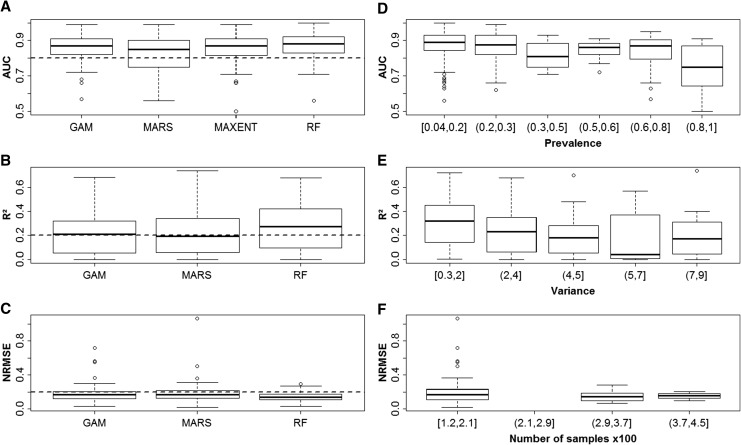
Performance of the different modeling methods for occurrence data (**a**) and abundance data (**b**, **c**) and importance of data traits: response prevalence (**d**), variance (**e**), and number of samples (**f**). Shown in *boxplots* are: *midpoint* median, *hinges* 25 and 75 % quantiles, and *whiskers* 1.5 times the spread (close to 95 % confidence intervals). *Dotted horizontal line* acceptable level of predictive accuracy or error

The performance of models was generally quite good across all taxa, study areas, and modeling methods (Fig. 1). The majority of the 292 occurrence models (55 %) were in the “fairly good” to “good” range with AUC values between 0.7 and 0.9. One-third of the models (31 %) achieved AUC values ≥0.9, which are considered “very good” (Hosmer and Lemeshow 2000). Of the 204 quantitative models, 16 % achieved NRMSE values below 0.1, corresponding to an average error of less than 10 % of the range of abundance values for the response. The majority of the models had NRMSE values ranging between 0.1 and 0.25. Values of *R* ^2^ reached a maximum value of 0.74, with 47 and 9 % of the models above 0.25 and 0.5, respectively. Model performance was consistent across all study areas, regardless of their differences in size (40–40 000 km^2^) or ranges of salinity, wave exposure, annual ice cover, and coastal morphology.

#### Modeling Methods

Overall, the performance in terms of observed AUC, *R* ^2^, and NRMSE was relatively similar among the methods (Fig. 1a–c). Nevertheless, models based on RF tended to be most accurate for both quantitative data and data on occurrence. RF was closely followed by GAM and MAXENT (only occurrence models), while MARS tended to be slightly less accurate. In general, predictions from RF resulted in a performance of AUC > 0.8 for models of occurrence and a precision of NRMSE < 0.2. Note also that even though the proportion of variance explained, *R* ^2^, was typically not higher than 0.15–0.40 for RF, the vast majority of these models was statistically significant, and thus they provide useful tools for formal quantitative predictions of cover, abundance, and diversity. Despite the fact that there were some systematic differences in performance among methods, the majority of methods predicted similar and consistent patterns of species distributions. Thus, we conclude that the spatial patterns of occurrence, abundance, and diversity of benthic species in the Baltic Sea can be successfully predicted using several nonlinear modeling techniques. Even though the performance of different methods is generally comparable, predictions produced by different methods could deviate slightly when projected into geographical space, which appeared to be related to both the spatial scale and the method-specific relative weights of the different predictors (Downie et al. 2013; Bergström et al. 2013; Sundblad et al. 2013). Therefore, we recommend an ensemble approach, integrating the results of several methods for mapping and for assessing uncertainties of spatial patterns (see also Araújo and New 2007; Grenouillet et al. 2011; Bergström et al. 2013; Sundblad et al. 2013 and references therein).

It is clear that there was a large variability in the performance of models among the different response variables within and among the methods (Fig. 1). Therefore, we also assessed how data traits such as response type, number of samples, sampling density, response prevalence of the occurrence data, and variance in the response abundance data affected model performance (Kadmon et al. 2003; Guisan et al. 2007; Li and Heap 2008). These analyses showed that prevalence was important in occurrence models explaining up to 31 % of variance of the model accuracy, with a higher AUC at a response prevalence <0.3 (Fig. 1d; see also Bučas et al. 2013). Variances in the response data and the number of samples were the most important factors for species abundance and diversity models explaining up to 36 % of variance in the predictive performance of the models. A higher predictive accuracy of abundance models could be achieved by reducing variance in the response data and increasing the sample size (Fig. 1e, f). Thus, it appears that data quality is an important issue for the performance of models, and consequently we recommend that sampling design for modeling should take into account the need to produce a comprehensive dataset that encompasses the appropriate environmental gradients within meaningful spatial scales for the modeled response.

#### Differences Among Types of Organisms

Despite some indications of consistently higher performance of models of macrophytes compared to those of invertebrates and fish, the main conclusion is that distributions of all investigated types of organisms can be predicted. However, there was a substantial variability in accuracy among taxa within these types of organisms. We tested whether this variability could be explained by the ecological traits of organisms (Downie et al. unpublished). It has been suggested that mobile, widespread, and generalistic species are difficult to model, as they are ubiquitous and show a very weak response to changes in the environment (McPherson and Jetz 2007; Syphard and Franklin 2009; Stokland et al. 2011). Our study partly supports this assertion, with significantly better predictive success in both occurrence and abundance models for epifauna and rooted plants, followed by macroalgae and infauna, and a poorer performance for fish.

#### Types of Predictor Variables

Our findings generally suggest that the explanatory power of various types of predictor variables was consistent across regional areas, although there were variations depending on the organism group considered (Gullström et al. unpublished). Environmental predictors important in quantitative models were also important in qualitative models. Bottom topography (primarily depth) and bottom substrate were generally the most powerful and important predictors, with strong effects on abundance and occurrence patterns of invertebrates and vegetation. For fish, geographic location and hydrographic variables (Secchi depth and salinity), tended to be more powerful predictors than depth and wave exposure, both regarding abundance and occurrence. Overall, the most striking conclusion is the vital role of detailed information on water depth and bottom substrate. Accordingly, access to high-resolution data on depth and substrate can greatly improve modeling and mapping, and is more or less a requirement for fulfilling the potential of predictive modeling of species distribution in benthic environments. An important message to any commissioning authority or other user is therefore that efforts to provide accurate high-resolution data on water depth and bottom substrate are needed to improve the quality of biodiversity maps.

#### Importance of Spatial Resolution

The predictive performance of empirical models is affected by uncertainty in the estimation of the response and predictor variables as well as uncertainties associated with the structure and formulation of a model. All of these sources of uncertainty are dependent on the spatial scale, i.e., extent and resolution. This means that the choice of sampling resolution can affect the accuracy of models and maps. In a specific study from the Swedish west coast using data collected in a hierarchical design, we assessed effects of spatial resolution on the predictive power of models of benthic flora and fauna (Svensson et al. 2013).

**Fig. 2:**
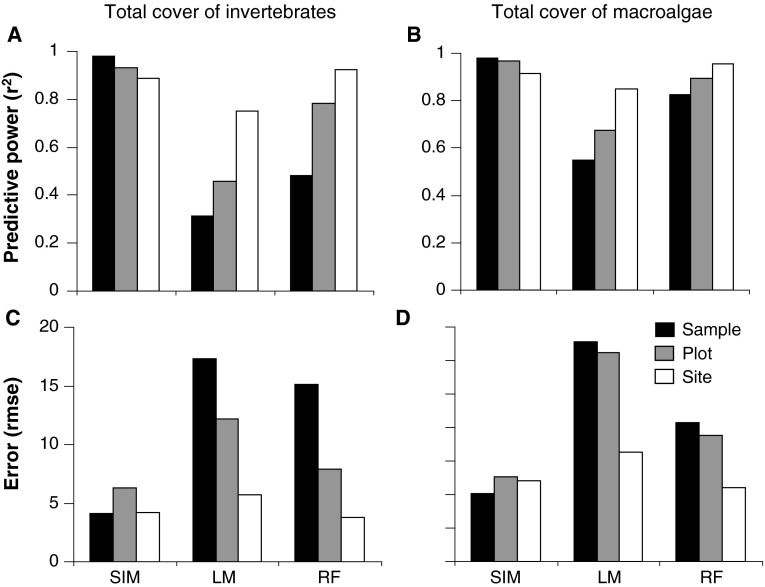
Comparisons of the simulated best achievable (SIM) predictive power (*r* ^2^) and precision (rmse) to those observed from a linear regression (LM) and a random forest (RF) model at three different resolutions: sample (1 × 1 m; *black*), plot (10 × 10 m; *gray*), and site (100 × 100 m; *white*) for cover of benthic marine invertebrates and macroalgae

In the study, we developed a simulation method to estimate the maximum achievable predictive power (*R* ^2^) and precision (RMSE) that would be expected based on uncertainty of estimates in the biological variables of interest. The precision and predictive power of these simulations were compared to the observed performance of a simple linear model (LM) and of a more flexible method (RF) (Fig. 2). Simulations showed that maximum predictive power and precision could be expected at fine resolutions (ca. 1 m). In contrast, the performance of quantitative models was better at relatively coarse resolutions (ca. 10 and 100 m). Hence, these analyses showed that based on sampling errors, model performance can often be expected to decrease at coarser resolutions due to larger spatial variability. In practice, however, the models often perform better at coarser or intermediate resolutions. The latter was not due to differences in sampling or spatial variability but is likely caused by a stronger mechanistic coupling between predictors (depth and hard substratum cover) and patterns at coarser scales.

Despite the potentially great impacts of resolution on model performance, studies addressing this issue are rare. Existing examples generally show better performance of models at fine resolutions (Graf et al. 2005; Heikkinen et al. 2007; Gottschalk et al. 2011). Thus, in contrast to both previous studies and error analyses we show that the finest resolution does not always result in optimum model performance and that aggregation at coarser spatial scales may instead be more efficient. Finally, by identifying the limitations imposed by lack of precise measurements at a certain scale, the methods developed also provide a tool for considering trade-offs between the need for more accurate measurements or for model refinement.

### Modeling Responses to Management Scenarios

One promising application of SDMs is using them to explore effects of alternative management scenarios relating to the conservation of species and habitats. While there are examples of studies that have used SDM to estimate future impacts of climate change on the distribution of species (e.g., Keith et al. 2008; Araújo et al. 2011), their potential for predicting effects of human pressures that can be managed at a regional scale has remained largely unexplored. The general approach is to develop empirical models where relevant human pressures, or proxies thereof, are used as predictors. An alternative method involves spatial overlay analyses between the predicted species or habitat and the pressure variable in combination with information on the change in distribution of the pressure variable over time. Within PREHAB, both these approaches were applied, in a case study on the effects of eutrophication and in another study on habitat exploitation through shoreline constructions.

In the study on eutrophication, potential ecological effects of eutrophication mitigation in accordance with the targets of the politically adopted BSAP were explored (Bergström et al. 2013). Despite the high economic costs involved in its implementation, effects on key species and habitats had not been assessed before. We explored the effects of changes in water clarity, measured as Secchi depth, a very important indicator of eutrophication status within the BSAP, on the distribution of key coastal species of perch (*Perca fluviatilis*), pikeperch (*Sander lucioperca*), eelgrass (*Zostera marina*), and bladderwrack (*Fucus vesiculosus*) in a 40 000 km^2^ archipelago area of the northern Baltic Sea.

**Fig. 3:**
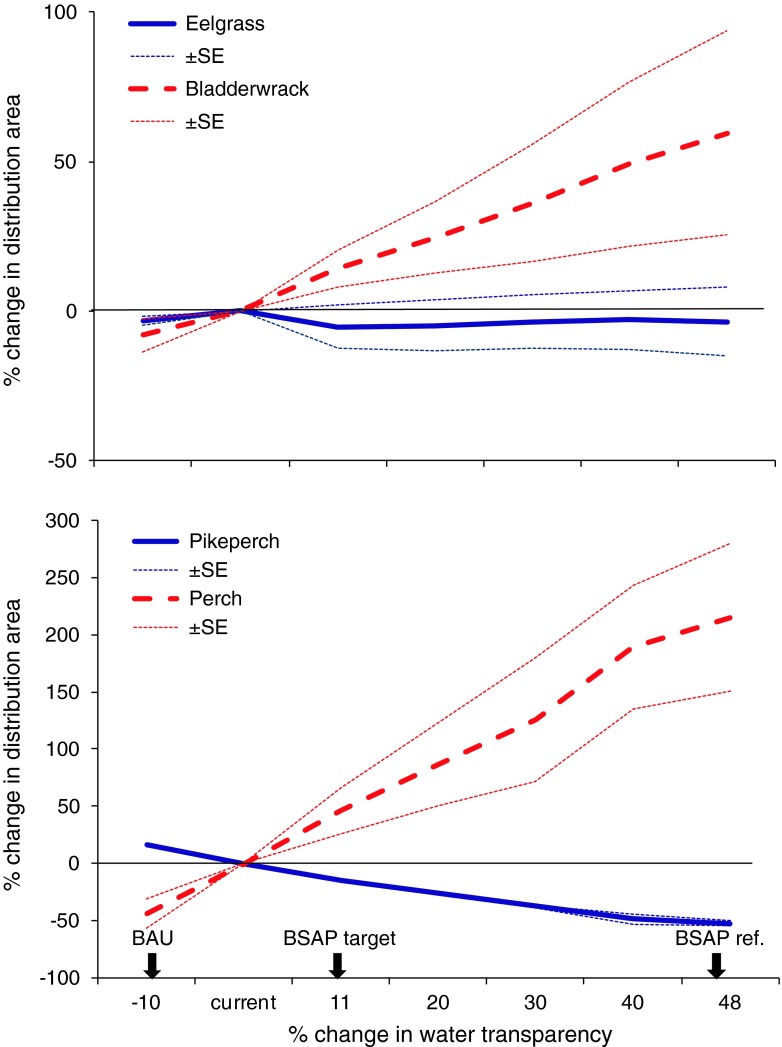
Predicted effects on the distribution of bladderwrack, eelgrass, and recruitment areas of perch and pikeperch as a response to changes in water clarity according to a set of management scenarios. *Dotted lines* are standard errors representing differences in predictions among three modeling methods. *Arrows* indicate the Secchi depth changes according to the scenarios business-as-usual (BAU), Baltic Sea Action Plan (BSAP) target level, and BSAP reference level (from Bergström et al. 2013)

Using an ensemble approach, three conceptually different methods (GAM, RF, and MAXENT) were compared to estimate effects of changes in water clarity on species distributions under a set of scenarios based on the BSAP. The three methods gave qualitatively similar results, although quantitative responses differed between them (Fig. 3). The analyses predicted that increasing water clarity, i.e., reduced eutrophication, would increase the distribution of bladderwrack, while the distribution of eelgrass remained largely unaffected. There would be a large increase in perch recruitment areas, and a concurrent decrease in recruitment areas of pikeperch. The different responses displayed by the species suggest that mitigation of eutrophication may have pronounced effects on ecosystem functioning by changing the simple food webs of the Baltic Sea. Despite the fact that water clarity is affected by other factors than the concentration of primary producers (Kratzer et al. 2004) and the uncertain efficiency of nutrient reductions as a means to improve water clarity, this study provides a step toward analyzing the ecological and economic consequences of the BSAP eutrophication objectives for the coastal ecosystem. Furthermore, the studied area is a substantial part of the central Baltic, but nevertheless, extrapolation to other areas or to the whole Baltic Sea needs to be done with caution.

The study of coastal habitat exploitation scenarios were used to examine consequences of long-term shoreline development (described in detail at www.prehab.gu.se). Maps of shoreline constructions in the form of jetties and marinas, which may affect habitats directly through building and dredging and indirectly through increased boating (Sandström et al. 2005), were combined with maps of predicted habitat distribution using spatial overlay analysis. The study showed that shoreline constructions have a strong local overlap with recruitment habitats for perch. Based on development rates from the 1960s and onwards (Kindström and Aneer 2007), our results indicated that around half of the recruitment habitats are currently exploited by shoreline constructions. The observed rates of construction differed between management areas, suggesting that current and future exploitation rates depend on policy and local management decisions. However, shoreline development is a slow process and it can be difficult to discover large system changes for management actions to avert negative regime shifts in time (Biggs et al. 2009), stressing the need to consider long-term cumulative impacts of small development projects.

**Fig. 4:**
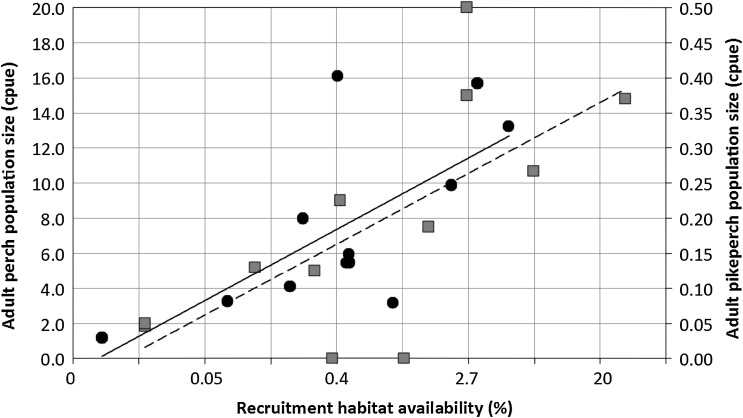
Adult fish population size as a function of recruitment habitat availability, within the average migration distance, for twelve populations of perch (*R* ^2^ = 0.46, *solid line* and *black circles*) and pikeperch (*R* ^2^ = 0.48, *dashed line* and *gray squares*) in the coastal areas of the Baltic Sea (modified after Sundblad et al. 2013). Note the ln-transformed *x*-axis

Assessing effects of human pressures on fish recruitment habitats becomes particularly important when considering population level consequences. We have also empirically demonstrated that key recruitment habitats can limit the size of adult populations of coastal fish (Sundblad et al. 2013). The study focused on perch and pikeperch, which are both ecologically and economically important in the Baltic Sea (Lehtonen et al. 1996; Eriksson et al. 2009). The study showed that almost half of the variation in population size could be explained by the availability of recruitment habitats. The relationships were nonlinear, suggesting that protection, or restoration, of habitats would have strongest effects in areas where there is currently little habitat available (Fig. 4). In addition, because the approach is spatially explicit, we identified areas where there is a particular need for habitat protection. By establishing a quantitative link between habitat distribution and fish population size we suggest that it is possible to estimate the potential production of adult fish, which is tightly coupled to economic values.

### Monetary Valuation of Management Scenarios

The contribution of economic analysis in PREHAB was to apply the results of predictive modeling in empirical policy analysis. The analysis supports sustainable coastal management and planning by providing monetary estimates of non-market benefits to be set against profits from economic use of marine ecosystems. Monetary estimates were gathered for predicted changes in key marine habitats associated with implementation of the BSAP in two Baltic coastal areas, the Finnish-Swedish archipelago and the Lithuanian coast (HELCOM 2007; Bergström et al. 2013; Sundblad et al. 2013). The valuation of a change means an assessment of the marginal benefits for a specified change (rather than the total value of benefits).

The economic valuation was based on a choice experiment. Choice experiments generally enable estimation of the value of ecosystem changes that have not yet taken place, and may be associated with non-use values, such as knowledge on the existence of marine ecosystems and species, and an option for future generations to enjoy ecosystem services provided by the marine environment. The idea of the method is to elicit citizens’ preferences through hypothetical market scenarios and to calculate the trade-offs between improvements in marine environment and monetary losses (see e.g., Champ et al. 2003).

Results of two choice experiment surveys conducted simultaneously in Finland, Lithuania, and Sweden, showed clearly that citizens in all three countries would value improvements in the preservation of currently “pristine” areas as well as in two ecosystem variables, habitat-forming vegetation and stocks of large predatory fish. Willingness-to-pay estimates for improvements in the marine environment differed significantly between countries (Kosenius and Ollikainen 2011). Calculation of national benefits from the implementation of the BSAP included three steps. First, the specification of the scenario (ecological impacts), second, the calculation of the associated average marginal benefits based on the coefficients estimated by a mixed logit model (Train 2009), and, finally, aggregation of these average values for the population in question. If the BSAP in the Swedish-Finnish archipelago results in a 50 % increase in the amount of healthy vegetation and fish stocks, compared to the situation in year 2010, the estimated aggregated benefit, as valued by the citizens, is €359 million for Finns and €1271 million for Swedes (Table 1). The Lithuanians value the same improvement in the Lithuanian coast to €30 million. Benefit/willingness to pay was not always linear, a twice-as-large change almost doubled the benefits in Finland (€659 million), while in Sweden and Lithuania the benefit was even more than twofold (€3501 and €79 million, respectively).

**Table 1:** Economic benefits (in € million) from increases in healthy vegetation and coastal fish stocks that might be a result of the implementation of the Baltic Sea Action Plan for three countries and selected coastal areas. The benefits, as perceived by the citizens in each country, are based on the mean willingness-to-pay estimates (Kosenius and Ollikainen 2011). Limits to 95 % confidence intervals in brackets

	Finland	Sweden	Lithuania
Sample/population	736/5375276	772/9408320	763/3329039
Benefit estimates in € millions			
Scenario 1: 50 % increase in healthy vegetation and fish stocks	359 (207–511)	1271 (786–1756)	30 (6–55)
Scenario 2: 100 % increase in healthy vegetation and fish stocks	659 (507–812)	3501 (2846–4153)	79 (55–102)

These estimates associate only to non-market benefits of selected coastal areas and selected populations. However, the improvement in the condition of the Baltic Sea benefits citizens in all Baltic Sea countries, and the valuation may not even be connected to the closest sea area. Therefore and additionally, due to large differences in average marginal benefit estimates in countries, we recommend these estimates to be used only in regional planning and not to be transferred to other Baltic Sea countries. For a Baltic-wide analysis, estimates related to the whole Baltic Sea would be more preferable (see Ahtiainen et al. 2013; BalticSTERN 2013). These analyses illustrate how SDMs, modeling of management scenarios, and valuation studies can be applied in a common framework to integrate landscape-scale ecological and economic impacts.

## Conclusions and Outlook

This paper summarizes the overall aims and results of the project PREHAB, which was funded during 2009–2011 under the BONUS+ program. Based on the results of the work, our overall conclusion is that habitat modeling and mapping is not only a promising, but a practically useful tool for addressing many of the challenges related to sustainable management and use of the Baltic Sea. Our main arguments for this conclusion are: (1) the general performance documented in our analyses across the Baltic Sea (Sundblad et al. 2011; Gonzalez-Mirelis and Lindegarth 2012; Bučas et al. 2013; Svensson et al. 2013, Sundblad et al. 2013), (2) the potential for integration of ecological and socio-economic systems demonstrated by the use of scenarios (Kosenius and Ollikainen 2011; Bergström et al. 2013), and (3) the continuing development of policies requiring new innovative tools for integrated assessments, e.g., the BSAP, MSFD, and proposed MSP directive.

First, the Baltic-wide synthesis of models suggest that models using species–environment relationships, derived from a range of statistical methods, can be used to predict both abundance and occurrence of vegetation, benthic invertebrates, and fish. Some differences in predictability can be explained by data quality and differences among taxonomic groups, but variability among species was generally unpredictable. Whether the performance of the models is sufficiently accurate for practical management depends on the demands of the management situation (e.g., Fielding 2002; Guisan and Thuiller 2005). Nevertheless, a discriminative power of AUC > 0.7 and 0.8 is generally considered “useful” and “excellent”, respectively, in scientific contexts (Hosmer and Lemeshow 2000; Maggini et al. 2006), and a relative error of NRMSE ≈ 0.20, characteristic for quantitative variables also appears useful. Furthermore, models performed well also with respect to distributions of functional groups and of important functional properties of the system (e.g., the abundance of primary producers and fish recruitment). Thus, there appear to be good opportunities for future predictions of important goods and services (e.g., Sanchirico and Mumby 2009).

Second, by combining information on human pressures and predictive models and by linking a study of economic valuation to different scenarios, we assessed ecological and economic benefits of a range of management scenarios (Kosenius and Ollikainen 2011; Bergström et al. 2013; Sundblad et al. 2013). While these kinds of predictions into new temporal domains have been used to forecast ecological effects of climate change and changes in land use, it is potentially associated with many types of uncertainties (Guisan and Thuiller 2005; Elith and Leathwick 2009; Fitzpatrick and Hargrove 2009). Nevertheless, the transformation of management targets formulated as a simple increase in water clarity, measured as Secchi depth, into estimates of ecological impacts in the geographic domain provided new and relevant perspectives for the management of the Baltic Sea, which would not have been possible without the empirical models. This includes illustrating conflicts between management objectives for different fish species, and for protection of fish recruitment habitats versus human shoreline exploitation. Considerations and solutions to such conflicts are increasingly important following implementation of ecosystem based management.

Third, the increasing pressures and impacts on the Baltic Sea environment require new tools for implementing more sustainable use and management. PREHAB was developed to contribute in some of these areas, and the results of these efforts have been formulated specifically for authorities and policy-makers in a user-friendly web-resource at www.prehab.gu.se. Nevertheless, the work toward sustainability is a continuing and adaptive process requiring cross-fertilization between policy and research. Some of the more prolific areas of interaction are those of marine spatial planning, linking structural and functional aspects of biodiversity, and integrated assessment of ecological and socio-economic systems (e.g., Crowder and Norse 2008; Backer and Frias 2013). A literature search in the ISI Web of Science showed that the number of scientific papers involving “marine spatial planning” and “species distribution model” in the title or abstract was 17 and 10 in 2009 when the project started. In 2012 when the project ended the corresponding numbers were 89 and 69, i.e., a more than fivefold increase in scientific interest. This strong development in the scientific domain is likely a result of the large applied needs for this kind of research and hopefully the results of PREHAB and similar efforts will be of increasing benefit for the future management of marine habitats globally and in the Baltic Sea.
